# Children’s informed signified and voluntary consent to heart surgery: Professionals’ practical perspectives

**DOI:** 10.1177/09697330211057202

**Published:** 2022-02-25

**Authors:** Priscilla Alderson, Hannah Bellsham-Revell, Joe Brierley, Nathalie Dedieu, Joanna Heath, Mae Johnson, Samantha Johnson, Alexia Katsatis, Romana Kazmi, Liz King, Rosa Mendizabal, Katy Sutcliffe, Judith Trowell, Trisha Vigneswaren, Hugo Wellesley, Jo Wray

**Affiliations:** Social Research Institute, 4919 University College, London, UK; Evelina Children’s Hospital, Guy’s and St Thomas’ NHS Trust, London, UK; Great Ormond Street Hospital NHS Trust, London, UK; 4956 Children’s Heart Federation, London, UK; Great Ormond Street Hospital NHS Trust, London, UK; Evelina Children’s Heart Organisation ECHO, London, UK; Little Hearts Matter, Birmingham, UK; Great Ormond Street Hospital NHS Trust, London, UK; Children’s Nursing, 4914 London South Bank University, London, UK; Social Research Institute, 4919 University College, London, UK; Tavistock Clinic London and University of Worcester, UK; Evelina Children’s Hospital, Guy’s and St Thomas’ NHS Trust, London, UK; Great Ormond Street Hospital NHS Trust, London, UK

**Keywords:** clinical ethics, informed consent, moral sensitivity, paediatric practice, truth-telling

## Abstract

**Background:**

The law and literature about children’s consent generally assume that patients aged under-18 cannot consent until around 12 years, and cannot refuse recommended surgery. Children deemed pre-competent do not have automatic rights to information or to protection from unwanted interventions. However, the observed practitioners tend to inform young children s, respect their consent or refusal, and help them to “want” to have the surgery. Refusal of heart transplantation by 6-year-olds is accepted.

**Research question:**

What are possible reasons to explain the differences between theories and practices about the ages when children begin to be informed about elective heart surgery, and when their consent or refusal begins to be respected?

**Research design, participants and context:**

Research methods included reviews of related healthcare, law and ethics literature; observations and conversations with staff and families in two London hospitals; audio-recorded semi-structured interviews with a purposive sample of 45 healthcare professionals and related experts; interviews and a survey with parents and children aged 6- to 15-years having elective surgery (not reported in this paper); meetings with an interdisciplinary advisory group; thematic analysis of qualitative data and co-authorship of papers with participants.

**Ethical considerations:**

Approval was granted by four research ethics committees/authorities. All interviewees gave their informed written consent.

**Findings:**

Interviewees explained their views and experiences about children’s ages of competence to understand and consent or refuse, analysed by their differing emphases on informed, signified or voluntary consent.

**Discussion:**

Differing views about children’s competence to understand and consent are associated with emphases on consent as an intellectual, practical and/or emotional process. **Conclusion**: Greater respect for children’s practical signified, emotional voluntary and intellectual informed consent can increase respectful understanding of children’s consent. Nurses play a vital part in children's practitioner-patient relationships and physical care and therefore in all three elements of consent.

## Introduction

Standards in the law and literature concerning children’s consent or refusal, summarized in the background section, differ from actual respectful practices in two London paediatric cardiology departments. After reporting the methods, this paper explores possible reasons for the contrast in the approaches to children’s consent. Thematic analysis of professionals’ interviews shows attention not mainly to informed consent, as in the literature, but also to signified and voluntary consent. The findings sections are divided between these three aspects of consent, which are then discussed. The conclusion considers their significance for healthcare practice, research and teaching.

## Background

There are clear differences between theories in the law and mainstream literature about children’s consent versus recently observed practices in two paediatric cardiac departments. Practitioners were observed to spend much time and effort on informing young children, responding to their resistance and refusal, and helping them to understand and ‘want’ the cardiac surgery. However, systematic reviews report that ‘shared decision-making is rarely implemented in paediatric practice’.^1^ Some reviews of consent to children’s surgery report only parents’ experiences and exclude children.^2^ The many meanings and methods of assent remain uncertain, and children’s dissent is largely ignored.^3^ It is widely assumed that legal minors cannot consent until they are around 12 years, and may not refuse recommended major treatment.^4^ Children who are deemed non-competent lack the automatic legal rights that protect most adult patients: to be informed and listened to, and not to be deceived or coerced. In 1998, a judge ordered that a 15 -year-old should be given a heart transplant against her will, showing that ‘the legal and ethical position on young people and consent is complicated and confused’.^5^

In contrast to assent, consent includes the choice to refuse, and has been clearly defined for decades.^6^ Though developed for medical research, the definitions of consent also apply to medical treatment and detailed definitions are considered in the Findings section. Numerous national and international professional healthcare guidelines emphasize the importance of consent as ‘essential’, though usually relying on parents’ consent.^7^ Most of the literature review is in the findings section, in order to show how an original three versions of consent analysis of the literature produces new findings, and to compare closely the main views in the literature with those in the hospitals.

## Research question

What are possible reasons to explain the wide differences between published theories and actual practices about the ages when children can begin to be informed about elective heart surgery, and when their consent or refusal begins to be respected?

### Research design, participants and research context

Research methods included reviews about children’s consent in the law, ethics and healthcare literature. During October 2019 to February 2020, there were ethnographic observations in two London paediatric cardiology departments in the wards, clinics and multidisciplinary meetings, and audio-recorded, semi-structured interviews, conducted with 45 healthcare professionals and related experts.^8^ The purposive multidisciplinary sample was interviewed once for about an hour in private face-to-face sessions October 2019 to February 2020, and then by phone between March 2020 and April 2021 Semi-structured question guides, based on previous research,^9^ asked about interviewees’ views and experiences concerning children’s heart surgery. The main questions considered in this paper concern the ages when interviewees consider children should begin to be informed about heart surgery, and when their consent or refusal should begin to be respected.

Encrypted interview recordings were professionally transcribed and then anonymized. Research with children aged 6- to 15 years having elective heart surgery and their parents was to be central to the project, but was much reduced because of COVID-19 when elective surgery was cancelled. This work is reported elsewhere. There were meetings with the interdisciplinary advisory group. Thematic data analysis was informed by the sociology of childhood studies where children are viewed as social agents with rights, in contrast to age-based developmental psychology.^10^ Grounded theory informed the repeated reading of the literature and research notes and transcripts to discover themes^11^ and attend to authors′ and interviewees′ emphasis on, or neglect of, three differing aspects of consent.

### Ethical considerations

Approval was granted by NHS HRA Research Ethics Committee (19/LO/0073), Hampstead Research Ethics Committee (ID 248332), and the Institute of Education University College London Research Ethics Committee (REC1188) in February 2019, and by the HRA-Confidentiality Advisory Group (19/CAG/0148) in September 2019. All interviewees gave their informed written consent and are anonymized. No patients were involved for this paper.

### Findings

During data analysis, gradually, different emphases on the three traditional major meanings of consent were noted. The consent law and literature highlight informed consent, central to law and the Declaration of Helsinki^12^: detailed intellectual understanding of the nature and purpose of the intervention, risks, benefits and alternatives. There is brief attention to signified consent when a legally authorized person signs the consent form. However, practitioners were very involved with practical signified consent, in children’s active cooperation or resistance. This is hardly mentioned in the literature, which also says little on voluntary consent in practitioner-patient relationships. Voluntary consent is essential and involves patients’ emotions of willing agreement and not feeling coerced,^8^ when trust and courage overcome fear and doubt. The findings are shown grouped into these three aspects of consent.

Table 1 shows the interviewees’ specialties. Table 2 summarizes the numbers of interviewees who cited each age when they thought children can begin to be informed, and to have their consent to heart surgery or their refusal respected. For example, 7 of the 45 interviewees would begin to inform children aged 2 years. The youngest age cited to begin informing children with a heart condition was ‘prenatally’ by interviewee 39. This is because information offered to parents and siblings after a prenatal diagnosis can profoundly shape their views of optimism or pessimism and thus may powerfully influence the affected child’s life, opportunities and sense of identity. Information givers should therefore take account of the unborn child’s perspective and future needs.

**Table 1.** Specialties of the 45 interviewees: members of the paediatric cardiac teams and related experts.

Specialty	Numbers of interviewees in each specialty. Some have two or three present or previous roles
Anaesthetists	5
Cardiologists	10
Chaplains	4
Children's heart charities support and information services	5
Ethics committee members	8
Intensivists	2
Lawyers	3
Mediator	1
Members of hospital directorates	5
Nurses	6
Paediatricians (not cardiologists or anaesthetists)	6
Palliative care (paediatric)	1
Patient care coordinator	1
Play specialists	2
Psychologists	4
Psychiatrist and psychoanalyst	1
Senior lecturer in nursing	1
Senior Operating Department Practitioner	1
Social worker for heart transplant families	1
Surgeons	3

^*^All doctors are consultants. All nurses have long experience and most have senior positions.

**Table 2.** Total numbers of experts’ views on the ages when children can begin to be informed, and when their consent to non-emergency heart surgery or their refusal begin to be respected.^5^

Age when the interviewees would begin	To inform the child	To respect the child’s consent	To respect the child’s refusal
Prenatal	1	—	—
0 or ‘from the beginning’ ‘as soon as possible’ ‘always’	5	—	1
1 year	1	—	—
2 years	7	—	1
3	7	1	2
4	5	3	1
5	2	1	3
6	7	7	4
7	2	5	1
8	—	5	4
9	—	1	—
10	2	5	2
11	1	3	1
12	—	3	3
13	—	—	—
14	—	1	1
15	—	—	—
16	—	2	2
17+	—	—	—
No reply/uncertain	5	8	18

#### Intellectual informed consent

The literature on consent is dominated by three authorities, which all set high ages for when legal minors can begin to give informed consent to medical treatment or research. There is the law, based on historical precedent,^4,13^ bioethics that draws on patriarchal philosophy and supports adult-centric reason and autonomy,^14^ and pharmaceutical companies running large trials that need rapid, legally valid consent systems.^15,16^ In healthcare and research, detailed informed consent has become a contract to defend against costly litigation,^17^ so that information should be as detailed as possible.^18^ The few published papers on children’s consent to treatment concentrate on those aged 12 years upwards, deemed in the (formerly British) Commonwealth *Gillick* competent,^19^ and in the US mature minors.^20^ Some lawyers are critical that ‘adolescent autonomy is little more than a myth’ and state that ‘no minor has a right to refuse treatment’.^4^ High standards of informed consent promote respect for adult patients and for parents, but leave children deemed pre-competent with no right to be informed, or involved in decisions. For children, consent is frequently replaced by the US concept of assent, which ‘is ill thought-out, confused and harmful’.^21^ Assent contradicts English *Gillick* law that children aged under-16 may consent, with no specified lower age, and assent need not be at all informed, but may be taken as agreement when children feel unable to refuse.^22^ Enforced treatment is then tacitly endorsed,^23^ despite official guidance to avoid coercion.^24^

Patients’ competence to consent is assessed by *a*) legal status, *b*) outcome (does the assessor consider the person’s decision is reasonable?) or *c*) function (even if the assessor disagrees with the patient’s decision does the reasoning that explains and justifies the decision seem valid?).^25^ Most adults are assessed as competent by status alone, whereas most children fail that test. They are then assessed for their reasoning (outcome, function) to high standards that many adults would fail, when *Gillick* competence involves ‘sufficient understanding and intelligence to understand fully what is proposed’.

The consent literature raises questions about informed consent: When can young children begin to understand their heart surgery? What do they need to know if their consent can be informed?

Theories of consent in the law and literature differ markedly from actual practice, observed in the two paediatric cardiology departments. Informing and listening to children are central to treatment from the early years. For certain complex high-risk operations, such as heart transplants, children’s consent is respected from 6 years. Their refusal may be respected at a younger age than their consent. From around 4 years, if they firmly resist, non-emergency surgery is postponed. Many interviewees, some of whom did not want to state a specific age (Table 2), described their respect for young children (Table 3).

**Table 3.** Interviewees’ views about intellectual informed consent.

3a ‘I find when I ask them people cannot recall information. I have had myself surgery and I do not recall anything’ (surgeon 15)
3b ‘[Two-year-olds] they can kind of, even at a really young age, understand this idea of something’s poorly and it needs some medicine or it needs to be fixed. And then that will help you feel better’ (psychologist 32)
3c ‘To explain techniques of single ventricle surgery to a 7-year-old is “probably overwhelming but saying, ‘We are going to make you feel so much less breathless, you are going to have so much more energy to play football,’ is more relevant” (cardiologist 14)
3d ‘We’ve talked to 3-year-olds and 4-year-olds about having a transplant and we’ve used analogies, so we’ve talked about cars and engines and they’ve understood… [In our research with children aged] 4 to 7 but also the children with learning disability… [on] things that they felt important about the hospital experience… they want people to look at them, they want people to answer their questions’ honestly and truthfully… I’ve been asked by children are they going to die, and I’ve had to be honest and say I don’t know if they’re going to die, I mean I certainly have never said, “No you are not”… People are recognising that we have to respect patients and value them and listen to them’ (psychologist 5)
3e ‘Children need to know what’s happening because I think they sense a lot of things. If they don’t know and no one explains things to them, they filter a lot of stuff and all they have is the anxiety and uncertainty without an explanation. That’s very distressing. Some parents are very reluctant about giving a lot of information to their children, so you need to find the balance between what parents are able to let you tell the child, what you feel comfortable to say to the child and what the child can understand’ (cardiologist 17)
3f ‘With ‘intervention for aortic valve stenosis, which could be a balloon [while the patient is conscious] or could be surgery… it’s very much down to understanding what the family’s attitude is to risk, what’s important to them’ (cardiologist 36)
3g ‘There’s often a lot of anxiety I think because of it being an unknown entity for children that haven’t been through the system before. And for children that have had surgery before, there’s anxiety related to memories of things that happened previously. A lot of it is around blood tests and cannulas and the pain aspect… rather than the pain aspect of surgery itself. I’ve also had patients that are very anxious about the anaesthetic, the idea of going to sleep and not waking up’ (cardiologist 21)
3h To respect a 6-year-old when she ‘definitely didn’t want [a heart transplant creates conflict] within a family because the parents don’t want to lose their child. [Yet] the compliance of the child is so pivotal that if they are not on board with it then you’ve wasted a huge resource’ (nurse 11)

Consent cannot involve understanding ‘fully’, the *Gillick* term, when it is given to partly unpredictable risks and futures. In paediatric cardiology, new knowledge and ‘far reaching’ change constantly emerge from research, experience and experiment.^26^ Doctors readily accept that their own knowledge is partial and fallible. The phrase, ‘I don’t know’, echoes through the interviews. They can tell even adult patients only a fraction of their specialized information, and find that adults and children have limited recall (Table 3a). Acknowledging experts’ partial knowledge encourages respect for young children’s limited though sometimes profound knowledge gained from experiencing serious illness and treatment (Table 3b–3d).

Reasons given for informing children include that they want and need to know what will happen to them (Table 3e),^27^ to correct misunderstandings and avoidable fears, and to understand that the purpose of surgery is well-intentioned, to help them (Table 3f). Traditionally, adults assumed they knew children’s ‘best interests’ and must override the objections of foolish, ignorant, potentially self-harming children.^28,29^ Recent guidance, however, challenges these assumptions advising that children should be ‘be kept as fully informed as they wish, and as is possible… to have their views and wishes sought and taken into account as part of promoting their welfare in the widest sense’.^24^ Interviewees repeatedly said that respecting children’s interests involves listening to their views about their interests and needs, to the extent of postponing non-urgent surgery to allow time to help children overcome their reasonable fears about risky painful interventions (Table 3g). At times the child’s choice is decisive, when there is a choice between surgery and an alternative intervention. With the scarcity of small hearts for transplantation, and if children still refuse after weeks of negotiation, donated hearts are reserved for informed, committed patients (Table 3h).

Those who cite high ages before minors can consent or refuse refer to informed consent as a legal contract, to the need for full details about the nature, purpose, procedures, risks, benefits and alternatives, and to the legal veto on minor’s refusal. Interviewees who cite lower ages tend to work more directly with young children informing them and, as far as possible with non-emergency treatment, respecting their consent or refusal. When preparing children for a heart transplant, during several in-patient days, clinical teams find that young children can have deep though not necessarily detailed understanding, that is respected as necessary and sufficient for consent or refusal.

#### Practical signified consent or refusal

The consent literature mainly portrays the passive patient with the active doctor who gives information and ‘does’ or even ‘provides’ the consents, expressions we frequently heard in the hospitals. However, doctors request but patients provide consent, or withhold it. Signified consent is usually seen as signing the consent form, which minors rarely do, although they are increasingly involved (Table 4a, 4b). Yet patients also signify consent or refusal when they cooperate with or resist interventions (Table 4c).

**Table 4.** Interviewees’ views about practical signified consent.

4a Signing the consent form for a heart transplant can help children aged from 8 years ‘to feel a bit of ownership about it [which] is good to have… as part of that process, alongside a parent’s signature’ (social worker 10)
4b ‘… 7- to 8-year-olds can be very involved… [not] as a sole decision maker, but you certainly can be involving them in the decision… We’ve designed a new consent form [showing] if it’s the child’s decision, then the parents can sign… saying they support [that or] where it’s the adult’s decision… the child can sign to say that they’re happy to go along with it’ (anaesthetist 18)
4c ‘There’s so much consent, you… come into hospital… get undressed and get into the bed… don’t like blood tests but you hold your arm out and allow that to happen… lots and lots of incidents like that… the small child has in a sense consented, in just the same way as an adult might… What you do not want is a sort of wrestling match where you sort of have to sit on them while you sedate them in order to proceed’ (surgeon 26)
4d Uninformed, unprepared children ‘tend to be very non-cooperative… very tearful and… anxious [and may be] terrified… When I’ve prepared them and explained things… you can see the change… [And] there’s been an uplift for the parents because sometimes [they have been afraid to inform their child].They’re smiling and they seem happier’ (play specialist 2)
4e When children from around 3 years ‘are mature in terms of illness [we need] to manage their expectations [so they can cooperate. They need to know] early on what their medicines are for, what their scars are about, why they might have another operation’ (psychologist 5)
4f ‘I have treated children after heart surgery who feel] panic, anxiety, depression, obviously fear and that’s partly real. I think the nightmares actually probably are the most alarming… One had a terrible one of some sort of cauldron fire thing and that was utterly terrifying. But just awful things happening’ (psychiatrist/psychoanalyst 41)
4g When young patients refuse life-saving heart surgery, ‘You’d have to look at the root cause… to see whether something has happened… look at their notes… Have they had a terrible experience with surgery before? Did they nearly pass?..Is there a reason that they are so adamant and negative towards this treatment? If [so]… some sort of therapy needs to be done to… get them to a place where they’re more accepting of what’s going on’ (children’s heart charity youth officer 34)

Resistance can express children’s need for more information and support. Interviewees reported that children ask more about procedures done while they are conscious, than about surgical techniques, so that they can understand and prepare for what they will experience. They can then signify their consent through informed cooperation (Table 4d, 4e). Practitioners often have to persuade protective parents to allow their child to be informed, and many described the dangers of overriding children’s resistance (Table 4f).

If children resist, interviewees advised taking time to calm them, to give more reassurance and information (Table 4g) combined as needed with some distraction, toys, books and videos to absorb the child, and some sedation before anaesthesia or lengthy procedures. Non-urgent surgery may be postponed, and nurses, play specialists and psychologists help the child to prepare to accept later surgery, assisted by imaginative programmes.^30–32^ Some practitioners advise replacing information with distraction techniques or medication to reduce pre-operative anxiety levels and distress.^33^ However, our interviewees used distraction and sometimes pre-operative medication to supplement but not replace discussions, and to support children’s informed cooperation. As one of the interviewees (with colleagues) stated publicly: ‘We often see children who are terrified, and have clearly suffered significant psychological trauma, having previously been held down at induction of anaesthesia by teams who did not mean to cause harm, but were just trying to get a procedure done. Rebuilding lost trust is a long and difficult process’.^34^

Negotiations with resisting children might seem to gently override children’s withholding of consent. Yet patients of all ages, who consent to the hoped-for benefits of surgery, may still fear the means towards achieving those ends: needles, incisions, scars, pain. Helping them to cope with distressing processes can respect their main decision. Lower ages of consent were cited by those closely involved with children’s practical signified consent.

#### Emotional voluntary consent

More relational concepts of children’s decision-making capacity have been advocated to increase practical respect for children,^35^ and these practitioner-patient, adult-child relational concepts are developed in this section. A frequent phrase is ‘young patients must consent’ to heart surgery. Yet that denies how consent can only be voluntary, freely given. Valid consent has long been identified with the rational person who calmly rises above emotions.^17^ Yet the *Nuremberg Code* emphasizes voluntary consent, feeling free not coerced.^8^ Consent involves trust, hope and belief in the benefits. ‘Belief’ originally meant ‘hold dear’, ‘consent’ meant ‘feel with’, and confidence meant ‘with faith’, all grounded in emotions.

Really understanding heart surgery involves some fear and dread (Table 5a) yet also knowing surgeons’ intention to benefit. Otherwise, children fear they are being punished and feel bewildered and guilty.^36^ Interviewees respected children’s emotions, and aimed to reduce their fear (Table 5b) and increase their confidence through trusting interpersonal relationships. This can take time (Table 5c). Children sense their parents’ anxieties, which alert them to the dangers they face, but potentially discourage them from expressing their own fears when they want to protect their parents (Table 5d). Practitioners encourage families to share their fears and hopes (Table 5e) and manage their emotions (Table 5f–5h). Children and parents need time to develop trust in the clinical team, courage to undertake risks, and hope for the benefits of surgery (Table 5i–5l). Doctors were observed warmly greeting babies, quietly explaining what they were doing while they examined them, and sensitively responding to babies’ moods and reactions. They would joke with older children about which football team they each supported. With new patients they have ‘to spend a lot of time’ working to establish trust' (cardiologist 16). ‘It’s a journey to build trust over years’ (cardiologist 17). Emotional voluntary consent and trust are motivations that give reality to consent however highly informed the patient might be.

**Table 5.** Interviewees’ views about emotional voluntary consent.

5a Four-year-olds ‘can’t understand every detail but they can understand that they have come in for an operation and they come in worried and stressed and anxious [and they] can tell you a lot’ (nurse 7)
5b ‘Any child who runs and hides, you should respond to that by engaging with them and exploring why. And that might not be an intellectual process all the time… [but] calming them down, reassuring them’ (ethicist 43)
5c ‘[Healthy teenagers] who suddenly collapse whilst playing football… [and receive a diagnosis and news about planned treatment] those kids they really need time… to digest those things… Generally, whatever is new you tend to refuse… but it’s the natural tendency of a person… the first reaction is either very curious or very scary… you can close off into yourself’ (cardiologist 14)
5d ‘Children are able to often, by 2, 3 [years of age], to understand other people’s emotions and see when someone’s crying and wants comfort. You know, it’s very, very common and very normal in hospital to have distressed parents and often they try and hide how they’re feeling. Kids pick up on it and you’ll see little children kind of wanting to hug their mums… to look after them… that’s just the dynamics within a family… that has experienced lots of distresses and uncertainty and concern about their child… And the child wants to protect parents, siblings, from further distress’ (psychologist 32)
5e The parents of an 8-year-old who did not want him to be informed were warned, ‘that if he knew absolutely nothing, when he came round from his surgery, he would be absolutely terrified, and so they reluctantly helped prepare this little boy for heart surgery. [We] worked alongside this family… [and after surgery] he wasn’t as terrified and the parents could see how important that was [after their] journey together. ‘The parents wrote a book for their son about what would happen to him. Like many others, one girl’ kept a lot of her fears to herself because she didn’t want to upset her parents.… Again, I think dying is something we don’t really talk about with children because it’s uncomfortable and very, very devastating’ (play specialist 25)
5f We help them ‘understand what’s going to happen, when it’s going to happen and why it’s going to happen [and they]… feel well equipped with a sense of choice, control and ability to cope’ (psychologist 20)
5g ‘We make massive efforts with the psychology team, with the anxiety management’ (surgeon 15)
5h ‘Amazing play specialists manage to get children who are really, really reluctant in the first instance, to get to a place where they actually let other people do something to them’ (cardiologist 17)
5i Trust helps children to feel: ‘You’re not a car that somebody’s just fixing, you’re a person that matters in this complex conversation’ (mediator, former paediatrician 35)
5j Intuitive trust is ‘not dependent on IQ, it’s dependent on the tone of voice [and one girl told me], “We’re not stupid, we tell from the tone of voice and the expression on the nurse’s and the doctor’s faces. Their eyes look sad” if there is bad news’ (nursing lecturer 42)
5k A 3-year-old after a heart transplant was supported by her mother ‘while the nurse searched for a vein and inserted a cannula [very calmly she] sat on her mother’s lap no bother… It was difficult, but it only took about 10 minutes [whereas] some parents talk about their own fear and spread so much negative energy around’ (nurse 7)
5l Children‘ are very perceptive and they are smart. They do notice things. I think it’s difficult to build trust and it’s difficult for them if the people they trust, like their parents or their doctor, are not honest with them and not completely open with them. I don’t think that’s right’ (cardiologist 17)

## Discussion

Differing views about children’s competence to understand and consent are associated with differing emphases on consent as an intellectual or practical or emotional process.

Beyond words on a form, signified consent exists through practitioner-patient interactions and voluntary consent exists within mutually respectful trusting relationships. They are as integral to consent as being informed. Actively involving children in signified and voluntary consent enabled practitioners to recognize their consent more fully. Yet the signified and voluntary elements are largely missing from the consent literature, though without them the nature and purpose of consent can become distorted. There may be undue emphasis on intellectual details that do not significantly alter the patient’s hope for the benefits of heart surgery or their courage to undertake the pain and risk. Consent may be diverted into becoming an abstraction instead of a relationship, serving the legal and financial protection of institutions rather than patients’ needs. Recognition that everyone’s knowledge about surgery is partial and fallible, and partly emotional as well as intellectual, helps to explain respect for children’s consent, when they basically apprehend risk and hoped-for benefit.

Children with congenital heart disease are unusual in that they tend to be highly aware and informed by years of illness and treatment. They depend on lifelong cardiac care. Relationships of informed trust in their healthcare professionals therefore matter far more to them, than they do to children having only brief treatment. Partially informed consent can be deceptive, misleading and can fail in the primary purpose of consent, to protect patients from needless dangers. Yet seemingly fully informed adult consent cannot guarantee full protection from misinformation or misunderstanding. The children in this research had the protections of their parents’ consent, and the weekly clinical team meetings concerned with evidence-based decisions about the best use of limited resources, as well as by both hospitals’ public records of heart surgery outcomes.

The study is limited to two leading hospitals and further research is needed in other centres and clinical specialties to see how these finding are, or could be, more generally applied. Topics briefly referred to in this paper, and missing topics such as assessing competence, are considered in other reports about this research.

## Conclusion

Younger children’s consent to heart surgery tends to be seen in the law and literature as neither necessary nor feasible. However, practitioners reported in research interviews that, more than assent, they respect children’s informed, signified and voluntary consent, three activities summed up by a surgeon^25^: ‘I don’t mean that the child should make the final decision and sign the form, but just make sure that the child understands and knows that this is best, and is willing to then go through the surgery and follow instructions afterwards’. This standard, from when children begin to be able and willing to understand and be involved in decisions, is seen by most interviewees as part of effective humane care. Because nurses play such a vital part in children’s physical care and relationships, they are especially aware of all three elements of consent.

The topic is important because, although heart surgery is unusual in being so complex and life-threatening, if young children can be informed and involved in these high-risk decisions, as many can, they suggest that children having many other less dangerous kinds of surgery can also be involved. Internationally, many children in middle-income and low-income countries cannot access much-needed surgical care. Yet, this paper is potentially relevant to all children receiving any kind of healthcare. It is about how healthcare professionals may inform and involve children far more than is often recognized, and encourage parents not only to represent their child, but also to involve their child as much as possible. Paediatric nurses play a vital part in children’s practitioner-patient relationships and physical care and therefore in all three elements of consent. Healthcare leadership, everyday practice, research and education need to be more informed by empirical research about the best current clinical practice, reported in this paper.
